# Editorial: Metabolic consequences of malnutrition: How to balance nutrients and genes

**DOI:** 10.3389/fnut.2022.1028502

**Published:** 2022-10-20

**Authors:** Hao-Yu Liu, Hui-Xin Liu, Ju-Sheng Zheng, Demin Cai

**Affiliations:** ^1^College of Animal Science and Technology, Yangzhou University, Yangzhou, China; ^2^Institute of Life Sciences, China Medical University, Shenyang, China; ^3^Key Laboratory of Growth Regulation and Translational Research of Zhejiang Province, School of Life Sciences, Westlake University, Hangzhou, China

**Keywords:** malnutrition, metabolic disorders, NAFLD, oxidative stress, dietary intervention, coronavirus, epigenetics

## Introduction

A healthy diet and exercise reduce the risk of chronic metabolic diseases. Nutrient intake and diet composition can have immediate and long-term beneficial or detrimental consequences on health. In this regard, maternal nutrition deficiencies may also impact the metabolic programming and health of the child. Malnutrition refers to imbalances of energy, protein, and other nutrients. Currently, the issues due to insufficient food availability have been largely overcome in developed countries. However, these countries begin to face the other side of malnutrition, i.e., overnutrition, which brought about high rates of chronic metabolic diseases. The interaction between nutrition, metabolism, and gene expression is crucial for the maintenance of whole-body homeostasis. The interplays between nutrition and the human genome can define and mark the gene expression and metabolic response. This in turn may affect the individual's health and susceptibility to disease. With the exponential increase of nutrition-related diseases, targeted approaches are needed to provide balanced diets in parallel with the development of national preventive health systems and screening programs adapted to local needs. Dietary intake is an essential factor, however, there is a marked inter-personal variation in metabolic disease onset, underpinning the significance of the complexity of interactions between genetic and environmental factors. This Research Topic “*Metabolic Consequences of Malnutrition: How to Balance Nutrients and Genes*” in Frontiers in Nutrition collected 14 scientific contributions from highly qualified research groups focusing on nutrition and metabolism-related diseases. This Research Topic is to clarify the basic knowledge about the vital role of nutrition-related genes in various disease states and to identify new concepts that could highlight the relation between nutrition and gene expression. Based on these studies, we hope to provide a better understanding of the mechanism and pathogenesis of metabolic disorders caused by a hyper-glucose, under-nourish, high-fat diet, micronutrient deficiency, oxidative stress, major depressive disorder, and even coronavirus diseases.

## Metabolic consequences of malnutrition

In particular, the nutrient-sensing nuclear receptor peroxisome proliferator-activated receptor-α (PPARα) is critical for the host response to short-term fasting and modifies the transcriptional programs of ketogenesis, fatty acid oxidation and transport, and autophagy in the liver. This regulation is ineffective in chronically undernourished individuals, often causing dyslipidemia and hepatic steatosis. Meanwhile, sirtuin-1 (SIRT1), a NAD-dependent deacetylase, is a regulator of nuclear receptors, decreasing their expression levels by proteasome-mediated degradation. Moore and Preidis' group revealed that PPARα is a novel target for Sirt1-mediated deacetylation, ubiquitination, and proteasomal degradation, and demonstrated that hepatic PPARα protein levels can be rescued in undernourished mice. These results pointed to a potential therapy targeting SIRT1 inhibition for undernutrition-induced liver and metabolic dysfunction (Suh et al.). Given SIRT1 is a mediator for AMPK-peroxisome proliferator activated receptor gamma coactivator 1-alpha (PGC1α) pathway, Zhang L. et al. revealed that the reduced AMPK-SIRT1-PGC1α signaling pathway caused by maternal high-fat diet (HFD), blunted the fatty acid β-oxidation in the placenta, leading to abnormal glucose and lipid metabolism of offspring at weaning. These findings indicate that fatty acid β-oxidation related gene profiles exert vital roles in modulating maternal overnutrition and metabolic health in the offspring (Zhang L. et al.).

In addition to metabolic diseases, overnutrition is also linked to intestinal inflammation. In a preclinical model of inflammatory bowel disease (IBD), Wu et al. have demonstrated that mice under short-term HFD exposure exhibited more severe clinical symptoms and colonic ulceration. In the intestinal mucosa of these animals, higher gene expressions of proinflammatory cytokines were observed (Wu et al.). In this study, the authors adopted new methodologies for identification and visualization of the spatial organization of bile acids (BAs) metabolism in the intestine of HFD-fed mice using mass spectrometry imaging (MSI). They demonstrated the application of MSI with a high spatial resolution (3 μm) plus mass accuracy matrix-assisted laser desorption ionization (MALDI) to identify BAs and N-1-naphthylphthalamic acid (NPA). This method could clearly determine the zonation patterns and regional difference characteristics of BAs on mouse liver, ileum, and colon tissue sections. The relative content of BAs based on NPA could also be ascertained (Zhang Q. et al.). In another mouse model of colitis, the core genes including nuclear factor (erythroid-derived 2)-like2 (Nrf2), signal transducer and activator of transcription 3 (STAT3), and phase II enzyme UDP-glucuronosyltransferase (UGT) were shown to be involved in the protective effects against colitis when treated with sulforaphane.

The effects of dietary interventions against malnutrition and inflammation are also explored. He et al. showed that an isothiocyanate, present in cruciferous vegetables such as broccoli and brussels sprouts, displays a therapeutic potential against ulcerative colitis. Nrf2 is one of the predominant factors to control oxidative stress by regulating the genes involved in the peroxidation pathway. Chen et al. found that oyster peptide (OP), a multi-nutritional food, substantially improves cyclophosphamide-induced intestinal oxidative stress in mice *via* the simulation of the antioxidant Nrf2-Keap1 signaling pathway. This study supports the potential application of peptide nutrients in the protection against oxidative stress and other metabolic disorders (Chen et al.). A review article summarized the important roles of branched-chain amino acids (BCAAs) in different metabolic disorders. BCAAs composed of leucine, isoleucine, and valine, account for about 35% of essential amino acids in most mammals and are critical in health and diseases. Lacking BCAAs may cause severe neurological disorders and growth retardation, while insulin resistance, obesity, heart failure, and even cancer are all associated with the accumulation of excess BCAAs. This review has also offered information about a series of novel methods for BCAAs measurement, facilitating researchers in the field to get a comprehensive picture of metabolic studies in BCAAs (Du et al.). It is worth mentioning that machine learning (ML) models have been successfully used in many obesity studies to predict obesity rates and identify risk factors in samples of interest. Because of that considerable current effort has been made by the computer science community and industry to apply artificial intelligence technology in the field of biology and biomedicine. A mini-review collected several ML algorithms or platforms, particularly focusing on nutrition, environment, and social factors, genetics or genomics, and microbiome research (Zhou et al.). This article also included detailed information on 23 open-source ML algorithms and related databases, including the project name, the linked website, applicable data types, and a simplified description of usage. ML algorithms are useful analytic tools that help us to conceptualize and study metabolic disorders within a fundamentally novel framework. So far, most ML algorithms used in metabolic research are in a single field. Whereas in the close future, the algorithms should be put together across platforms or data types.

Fructose is a pivotal nutrient that has been suggested to be strongly linked to metabolic diseases. Low dietary fructose increases the length of the intestine and the height of intestinal villi, contributing to weight gain and fat accumulation, which suggests the potential and beneficial role of fructose with appropriate concentration. Animal models are widely employed for studying fructose-induced metabolic changes. The anatomical and physiological similarities between pigs and humans suggest that a pig model is an important tool for biomedical research. In addition, pig production plays an important role in farming systems worldwide and the large-scale pig industry is developing rapidly all over the world to satisfy the requirement of growing consumers. The application of fructose in pig biomedical models and pig production needs further progress to production methods majorization and study of human diseases more accurately. A mini-review concluded the characteristics and metabolism of fructose in pig reproduction, growth, development, and as a human biomedical model (Xie et al.). This review addresses fructose metabolism in several key organs and its related functions in the intestine and blood cycle, benefiting new strategies for maintaining blood glucose balance and curbing metabolic diseases in humans and animals. Disagreeing with the existing *t*-test of a correlation coefficient, Aslam and Albassam provide a new way of investigating the relationship between fasting blood glucose level and drinking glucose solution. In this perspective article, the authors performed the test on data obtained from diabetes patients. The proposed method of a correlation coefficient was demonstrated to be effective for studying the significance of correlation in an indeterminate environment. It is anticipated that this new method could be applied to investigate correlations in a wide field involving economics, business, medicine, and industry in future. Till today, anti-diabetic oral agents and nutritional management are frequently used together as first-line therapies for type 2 diabetes mellitus (T2DM). However, their interaction is still unclear. Serving as the classic anti-diabetic medications, the interactive effect of acarbose and metformin with dietary intakes of macronutrients on glycemic control and cardiometabolic risk factors in a Chinese cohort was investigated as the initial hypoglycemic treatment (MARCH) randomized clinical trial (An et al.). Metformin and acarbose mainly exerted divergent interactive effects with dietary macronutrients on GLP-1 secretion, insulin release, and SBP, implying the distinct benefits for glycemic control due to the complexities of drug-diet therapies. The novel findings from the MARCH trial highlight the complicated nature of combining drug and diet therapies, and concomitant use of drug and diet with an expectation of additive may further benefit different hypoglycemic medications.

Higher adiposity is tightly linked to the aggravation of COVID symptoms, and the white adipose tissue (WAT) exerts a positive response to the infection by SARS-CoV-2. Moreover, adipokines like adiponectin are demonstrated to be associated with lipid metabolism and inflammatory factor secretion in patients with SARS-CoV-2. This adipokine may thus influence COVID-19 severity directly and indirectly. In a prospective study by Minuzzi et al., 145 hospitalized patients with COVID-19 were evaluated. They showed a robust linkage between brain-derived neurotrophic factor (BDNF) levels and COVID-19 severity (Minuzzi et al.). While adiponectin and leptin do not predict disease severity, the ratio of BDNF/adiponectin is informative of patient status and is sex-specific. These results reveal that serum BDNF content and BDNF/adiponectin ratio may serve as tools for predicting worsened prognosis in COVID-19, especially for male patients. Given that cholesterol is required for coronavirus infection *in vitro*, the role of endogenous cholesterol metabolism in regulating coronavirus infection and the mechanism behind it should be elucidated. With a mechanistic study in an animal model, Liu et al. demonstrate that a porcine coronavirus triggers an aberrant regulation of cholesterol metabolic genes *via* epigenetic inhibition of SREBP2/FXR-mediated transcription, offering a novel antiviral strategy against PEDV and other coronaviruses. Since the outbreak of COVID-19, numerous studies from around the world have reported declines in mental health such as major depressive disorder (MDD), which is a complex, multifactorial disorder of rising prevalence and incidence worldwide. The link between nutritional epigenetics and MDD composes a new field of research. A deep understanding of these diet-related epigenetic shifts becomes necessary highlighting complementary branches such as nutritional neuroscience and nutritional psychology for the integrative study of MDD. Thus, Ortega et al. present a critical review to integrate different areas of research to serve as a link between malnutrition-related epigenetic changes involved in MDD pathophysiology. They have discussed metabolic changes derived from an impairment in cellular processes owing to lacking some essential nutrients in the diet and therefore in the organism. Finally, aspects related to nutritional interventions and recommendations are also discussed.

## Conclusions and perspectives

The epigenetic actions of nutrition suggest that it can modulate numerous metabolic pathways which have been investigated in the context of metabolic disorder pathophysiology. Novel advances in clinical trials have generated promising results in the ability of nutrition intervention to reverse or attenuate these epigenetic marks. There are still large gaps in the understanding of the pathophysiology of metabolic diseases and the associated epigenetic drivers, signature genes, and functional pathways, even more in the knowledge of the connection between malnutrition and consequent epigenetic marks involved in metabolic disease pathophysiology.

## Author contributions

DC wrote the introduction and the conclusion. H-YL wrote the central part with comments on the cited papers and references. H-XL and J-SZ contributed to the review and editing. All authors contributed to the article and approved the submitted version.

## Funding

This work was supported by the Natural Science Foundation of Jiangsu Province (BK20200932 and BK20220582), Natural Science Foundation of the Higher Education Institutions of Jiangsu Province (20KJB230001), and the Priority Academic Program Development of Jiangsu Higher Education Institutions (PAPD).

## Conflict of interest

The authors declare that the research was conducted in the absence of any commercial or financial relationships that could be construed as a potential conflict of interest.

## Publisher's note

All claims expressed in this article are solely those of the authors and do not necessarily represent those of their affiliated organizations, or those of the publisher, the editors and the reviewers. Any product that may be evaluated in this article, or claim that may be made by its manufacturer, is not guaranteed or endorsed by the publisher.

